# The effect of ultrasound-guided lung recruitment maneuvers on atelectasis in lung-healthy patients undergoing laparoscopic gynecologic surgery: a randomized controlled trial

**DOI:** 10.1186/s12871-022-01742-1

**Published:** 2022-07-01

**Authors:** Yi Liu, Jingyu Wang, Yuan Geng, Yiran Zhang, Hang Su, Yujiao Yang

**Affiliations:** 1 Department of Anesthesiology, Chengdu Second Peoples Hospital, Chengdu, 610021 Sichuan Province China; 2 Department of Anesthesiology, Affiliated Hospital of North Sichuan Medical College, Nanchong, 637000 Sichuan Province China; 3 Department of Nephrology, The First Affiliated Hospital of China Medical University, Shenyang, 110000 Liaoning Province China

**Keywords:** Lung ultrasound, Atelectasis, Recruitment maneuvers, PEEP

## Abstract

**Background:**

Atelectasis is the primary cause of hypoxemia during general anesthesia. This study aimed to evaluate the impact of the combination of recruitment maneuvers (RM) and positive end-expiratory pressure (PEEP) on the incidence of atelectasis in adult women undergoing gynecologic laparoscopic surgery using pulmonary ultrasound.

**Methods:**

In this study, 42 patients with healthy lungs undergoing laparoscopic gynecologic surgery were randomly divided into the recruitment maneuver group (RM group; 6 cm H_2_O PEEP and RM) or the control group (C group; 6 cm H_2_O PEEP and no RM), 21 patients in each group. Volume-controlled ventilation was used in all selected patients, with a tidal volume of 6–8 mL·kg^−1^ of ideal body weight. When atelectasis was detected, patients in the RM group received ultrasound-guided RM, while those in the C group received no intervention. The incidence and severity of atelectasis were determined using lung ultrasound scores.

**Results:**

A total of 41 patients were investigated. The incidence of atelectasis was lower in the RM group (40%) than in the C group (80%) 15 min after arrival in the post-anesthesia care unit (PACU). Meanwhile, lung ultrasound scores (LUSs) were lower in the RM group compared to the C group. In addition, the differences in the LUS between the two groups were mainly due to the differences in lung ultrasound scores in the posterior regions. However, this difference did not persist after 24 h of surgery.

**Conclusions:**

In conclusion, the combination of RM and PEEP could reduce the incidence of atelectasis in patients with healthy lungs 15 min after arrival at the PACU; however, it disappeared within 24 h after surgery.

**Trial registration:**

(Prospectively registered): ChiCTR2000033529. Registered on 4/6/2020.

**Supplementary Information:**

The online version contains supplementary material available at 10.1186/s12871-022-01742-1.

## Background

Atelectasis is a common mechanical ventilation complication that contributes to the development of postoperative pulmonary complications (PPCs) [1]. The incidence of atelectasis after general anesthesia is up to 90% [2], and it can occur during induction of anesthesia and last up to 2 days postoperatively. Furthermore, atelectasis is the primary cause of hypoxemia during general anesthesia [3].

The definitive pathophysiological mechanism in the development of atelectasis remains unknown. Absorption, compression, and reduction in surfactant are the most consistent mechanisms [4]. Patients undergoing gynecological laparoscopic surgery should often maintain the carbon dioxide pneumoperitoneum and Trendelenburg position, and this can increase the probability of atelectasis and the possibility of hypoxemia, which promotes PPC [5, 6].

The use of protective intraoperative mechanical ventilation has been associated with a lower incidence of atelectasis and PPC. Although positive end-expiratory pressure (PEEP) alone can improve intraoperative oxygenation [7], studies have shown that the combination of recruitment maneuvers (RM) and PEEP can improve oxygenation and reduce the incidence of PPCs in patients better than either alone [8–12]. However, when low tidal volumes were used in various ventilator strategies, increasing PEEP with alveolar recruitment maneuvers did not reduce the incidence of postoperative pulmonary complications, as observed in some large studies with large sample sizes [13–15]. Until now, it remains unclear whether low levels of PEEP combined with recruitment maneuvers can reduce PPCs compared to low levels of PEEP alone.

Lung ultrasound (LUS) is a simple bedside imaging method that is less expensive, portable, and noninvasive compared to CT, the gold standard for the clinical diagnosis of atelectasis. It allows the repetition of multiple simple and seriated exams to compare and follow the state of the lung, and it is especially useful in cases of limited mobility as named exams. Furthermore, pulmonary ultrasound can be used to diagnose and monitor atelectasis accurately [16–19]. For these reasons, we assessed how lung ultrasound-guided RM combined with PEEP affect the incidence of atelectasis in lung-healthy adult female patients undergoing gynecological laparoscopic surgery. We hypothesized that lung ultrasound-guided RM combined with PEEP would reduce the incidence of atelectasis in lung-healthy patients undergoing gynecologic laparoscopic surgery.

## Methods

### Study design

From June to October 2020, a prospective randomized controlled study was carried out. The Ethics Committee of the Affiliated Hospital of the North Sichuan Medical College approved the study (Ethical No. 2020ER079–1). All participants signed an informed consent form and registered with the China Clinical Trials Center (Approval No. ChiCTR2000033529, registration date: 4/6/2020). All procedures followed the relevant CONSORT guidelines.

### Study population

The inclusion criteria were as follows: patients with healthy lungs (negative imaging findings on chest radiograph and CT) who were between 18 and 65 years old, with a body mass index (BMI) < 35 kg / m2, physical status I-II of the American Society of Anesthesiologists (ASA) and undergoing elective gynecologic laparoscopic surgery (benign neoplasms and precancerous lesions). Patients with pulmonary, cardiac, and neuromuscular diseases and a corresponding surgical history and respiratory tract infections were excluded. The following patients were also excluded: (1) those with preoperative ultrasound evidence of pulmonary atelectasis; (2) those undergoing conversion from laparoscopic to open surgery; and (3) those experiencing critical postoperative complications such as severe subcutaneous emphysema and pneumothorax.

### Randomization and blinding

According to computerized randomization software, patients were randomly assigned to one of two groups: control (C) or recruitment maneuvers (RM) (www.randomization.com). These tasks were hidden in sealed envelopes that were opened after the anesthesiologist had administered general anesthesia to a patient. An anesthesiologist performed anesthesia management and ultrasound, while a radiologist performed ultrasound scoring. Only the anesthesiologist performing the anesthesia induction and the pulmonary ultrasound was aware of the grouping details; the patient or the pulmonary ultrasound evaluator (a professional imaging doctor) had no such knowledge.

### Anesthesia and ventilation protocol

All patients received the standard general anesthetic protocol. This includes mask ventilation with pure oxygen at 5 L·min^−1^ for 3 min, induction of 0.04 mg·kg^−1^ midazolam, 0.5 µg·kg^−1^ sufentanil, 2 mg·kg^−1^ propofol, and 0.6 mg·kg^−1^ rocuronium, and use of the appropriate size of the tracheal tube for intubation. Using Dräger Fabius plus XL, the volume-controlled mechanical ventilation mode was performed after intubation with a tidal volume of 6–8 mL·kg^−1^ of ideal body weight, PEEP of 6 cmH_2_O, and 0.4 inspired oxygen fraction (FIO_2_). The ideal body weight (IBW) was calculated according to the following predefined formula for women: IBW (kg) = 45.5 + (0.91 × [height in centimeters − 152.4] [20].

The initial respiratory rate was set at 12 breaths per minute, with a 1:2 inspiratory: expiratory ratio. The ventilator was set to keep the end-tidal carbon dioxide pressure between 35 and 45 mmHg. Anesthesiologists could adjust FIO_2_ when peripheral oxygen saturation reached 90%, according to their experience. The Trendelenburg angle was set to 30°. Anesthesia was maintained with intravenous infusions of 0.1–0.3 µg·kg^–1^·min^–1^ remifentanil, 4–12 mg·kg^–1^·h^–1^ propofol, and inhalation of 1 to 3% sevoflurane. Bispectral index monitoring was used to monitor and maintain the depth of anesthesia at 40–60. To maintain adequate muscle relaxation, the timely supplement rocuronium was used. After spontaneous breathing recovery, neuromuscular blockade was reversed with neostigmine (0.05 mg·kg^–1^) and glycopyrrolate (0.007 mg·kg^–1^). Tracheal extubation was permitted only after adequate neuromuscular function was achieved (as documented by a measured train-of-four ratio of more than 0.90). After extubation, the patient was transferred to the post-anesthesia care unit (PACU) and oxygen was administered by nasal cannula inhalation at a flow rate of 3 L·min^–1^. A numerical pain rating scale was used to assess postoperative pain (NRS). Pulse oxygen saturation (SpO_2_), mechanical ventilation time, PACU stay time, NRS, PPC, and hospital stay time were recorded.

### Lung ultrasonography

An ultrasound machine (MINDRAY M9) with a probe of 2–5 MHz was used by a trained and experienced anesthesiologist to perform the lung ultrasound. Sonograms were taken at five predetermined times: when the patient entered the operating room (time point 1, T1), 1 min after mechanical ventilation (time point 2, T2), at the end of surgery before extubation (time point 3, T3), 15 min after arrival in the PACU (time point 4, T4), and 24 h after surgery (time point 5, T5). Scanning was carried out in the manner described by Monastesse et al. [18].

**Fig. 1:**
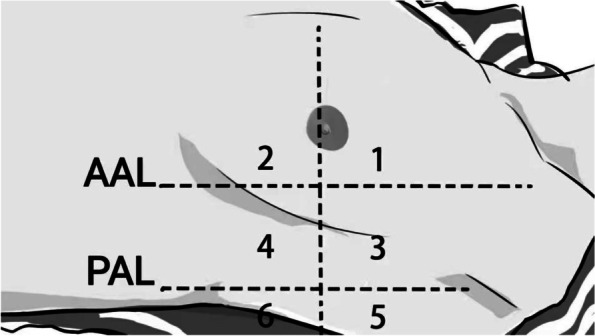
Each hemithorax was separated into 6 quadrants: anterior, lateral and posterior zones separated by the anterior and posterior axillary lines as anatomical landmarks, and each area was further divided into superior and inferior portions. AAL – anterior axillary line; PAL – posterior axillary line

Each hemithorax was divided into two zones: upper and lower, and each side was further divided into anterior, lateral and posterior zones by the anterior and posterior axillary lines. As shown in Fig. 1, each hemithorax is divided into six quadrants for a total of twelve quadrants. In the anterior and lateral regions, the probe was placed upright to the costal space, whereas in the posterior regions, the probe was placed parallel to the intercostal space. The modified lung ultrasound score developed by Monastesse et al. was used to quantify the severity of atelectasis [18].

The lung ultrasound score (LUS) was independently assigned by a radiologist and was ranked on a four-point scale. The scoring ranged from 0 to 3, as follows: (1) ≥ 3 B lines or one or more small subpleural consolidations separated by a normal pleural line; (2) multiple coalescent B lines or multiple small subpleural consolidations separated by a thickened or irregular pleural line; and (3) consolidation or small subpleural consolidation of > 1 × 2 cm in diameter. Atelectasis was considered significant if the LUS ≥ 2 was present in any region. LUS was determined by adding the scores of the 12 individual quadrants, ranging from 0 to 36 points, with higher scores indicating a more severe loss of aeration.

### Study protocol

When atelectasis was detected using ultrasound after 1 min of mechanical ventilation and at the end of the surgery, patients in the RM group underwent lung recruitment. The probe was placed in the atelectasis area, and the ventilator pressure parameters were adjusted by the operator. Furthermore, the maximum airway pressure was set to start at 10 cmH_2_O and gradually increased by 5 cmH_2_O until the collapsed lung area was not visible on ultrasound. The current pressure was kept constant for 40 s, with a maximum airway pressure not exceeding 40 cmH_2_O. Mean arterial pressure and heart rate were modified < 15%. When the decrease in blood pressure was greater than 20% of the baseline value or the SBP was reduced to 80 mmHg, ephedrine 6–10 mg was administered immediately. When the heart rate was less than 50 beats per minute, 0.3–0.5 mg atropine was administered.

### Perioperative observations

Age, BMI, ASA classification, type of surgery, total fluid intake/output, operating time, pneumoperitoneum CO_2,_ and NRS were all evaluated as baseline characteristics. At the above five time points, the observation indices were LUS score and SpO_2_, fluid volume, mechanical ventilation, PACU residence and hospitalization time, and PPC.

### Primary and secondary endpoints

The primary endpoint was the appearance of atelectasis, with secondary endpoints, including the LUS score, oxygen saturation, PACU residence time, hospitalization time, and PPCs.

### Sample size estimation

The sample size was calculated using the data from the previous studies. Yang et al. found that the frequency of atelectasis after a lung recruitment maneuver was 50%, compared with 95% in adults after laparoscopic colorectal surgery who did not receive a recruitment maneuver [21]. The sensitivity of lung ultrasound to detect atelectasis is 88% [19]. In our preliminary study, the incidence of atelectasis was 81.8% in patients with healthy lungs after gynecologic laparoscopic surgery, 15 min after arrival in the PACU, and was reduced to 40% using RM. As a result, PASS 15 calculated the required sample size to be 20 patients per group, assuming an alpha error of 0.05, a power of 80%, and a dropout rate of 10%.

### Statistical analysis

The demographic and anthropometric data of the individual patients were collected. Data were normalized using the Shapiro–Wilk test. For data with a normal distribution, the t-test or repeated measurement analysis of variance was used; for nonnormally distributed data, the Mann–Whitney U test or Cochran Q test was used. For categorical variables, the Chi-square or Fisher’s exact test was used. Unless Bonferroni adjustments were made, a two-sided *p*-value < 0.05 was considered significant. For statistical analyses, SPSS 25.0 and GraphPad Prism 8 software were used.

## Results

**Fig. 2:**
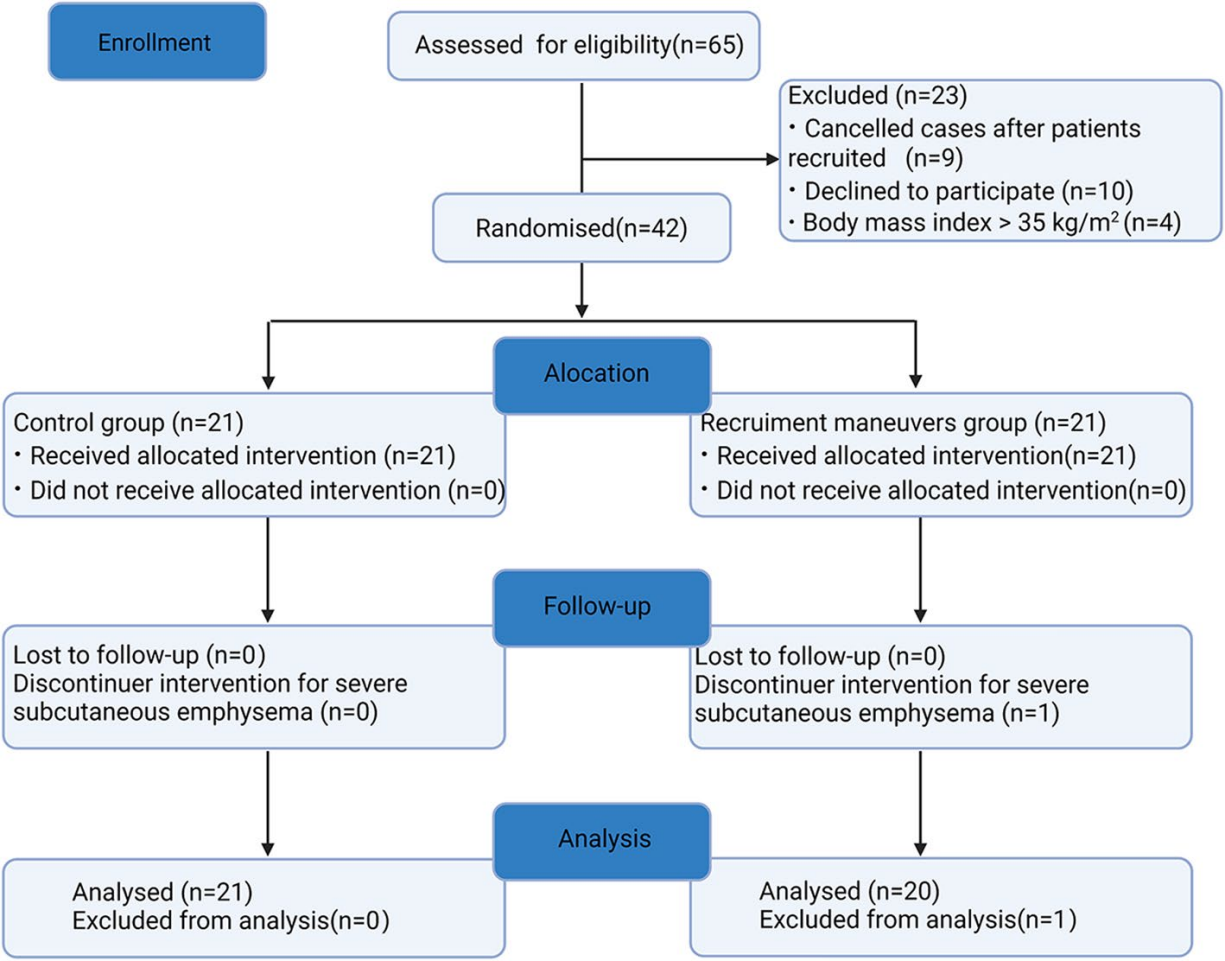
Flow diagram of patient screening and enrollment

A total of 65 patients were included in this study from June to October 2020. Among these, 23 patients were excluded for different reasons, as illustrated in Fig. 2.

**Fig. 3:**
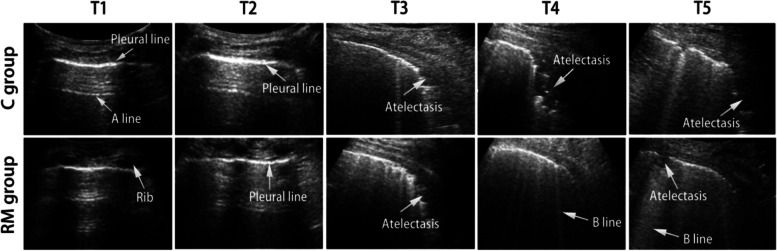
Lung ultrasound images of one representative patient per group

The remaining 42 patients were randomly assigned to one of two groups: RM or C. One patient in the RM group dropped out due to severe postoperative subcutaneous emphysema, which resulted in poorly visualized lung ultrasound. Finally, 21 and 20 patients in the C and RM groups, respectively, were included in the analysis (Fig. 2). A total of 2460 images were collected, and Fig. 3 shows representative lung ultrasound images at various times.

Table 1 shows the baseline characteristics of the patients included in the study. There were no significant differences, and all participants had no significant comorbidities or smoking status.

**Table 1:** Baseline characteristics

	C group, *n* = 21	RM group, *n* = 20	P*
Age (years)	38.1 ± 10.9	37.2 ± 9.7	0.759
BMI (kg/m^2^)	21.6 ± 3.1	22.0 ± 2.5	0.690
ASA classification (I/II)	0/21	2/18	0.142
Type of surgery (n)			
Laparoscopic total hysterectomy (n)	10	9	0.867
Adnexectomy (n)	6	5	0.796
Ovarian cyst removal (n)	2	2	1.000
Tubectomy (n)	3	4	0.697
Total fluid intake (ml)	1600.0(1100.0–1700.0)	1600.0(1100.0–1600.0)	0.619
Total fluid output (ml;)	230.0(200.0–485.0)	300.0(162.5–465.0)	1.000
Operating time (min)	84.9 ± 33.2	86.9 ± 37.1	0.857
CO_2_ pneumoperitoneum (mmHg)	13.0(12.0–14.0)	13.0(12.0–14.0)	0.739
Postoperative pain (numerical pain rating scale)			
T4	1.9 ± 1.3	2 ± 1.3	0.724
T5	1.3 ± 1.2	1.2 ± 1.2	0.822

Data are presented as the mean ± standard deviation unless otherwise indicated.

* Comparison between the two groups per time point, with *P* < 0.05 considered significant

At each time point, the incidence of atelectasis in both groups is shown in Supplementary Table 1. There was no statistical difference in the incidence of atelectasis between the two groups at T2, and this remained true until after the first RM. When observed at T4, the incidence of atelectasis in the RM group was lower than that in the C group after the second RM, and this difference vanished within 24 h of surgery (T5). Furthermore, no PPCs were observed in either group, and no side effects of recruitment maneuvers were observed in the RM group.

**Fig. 4:**
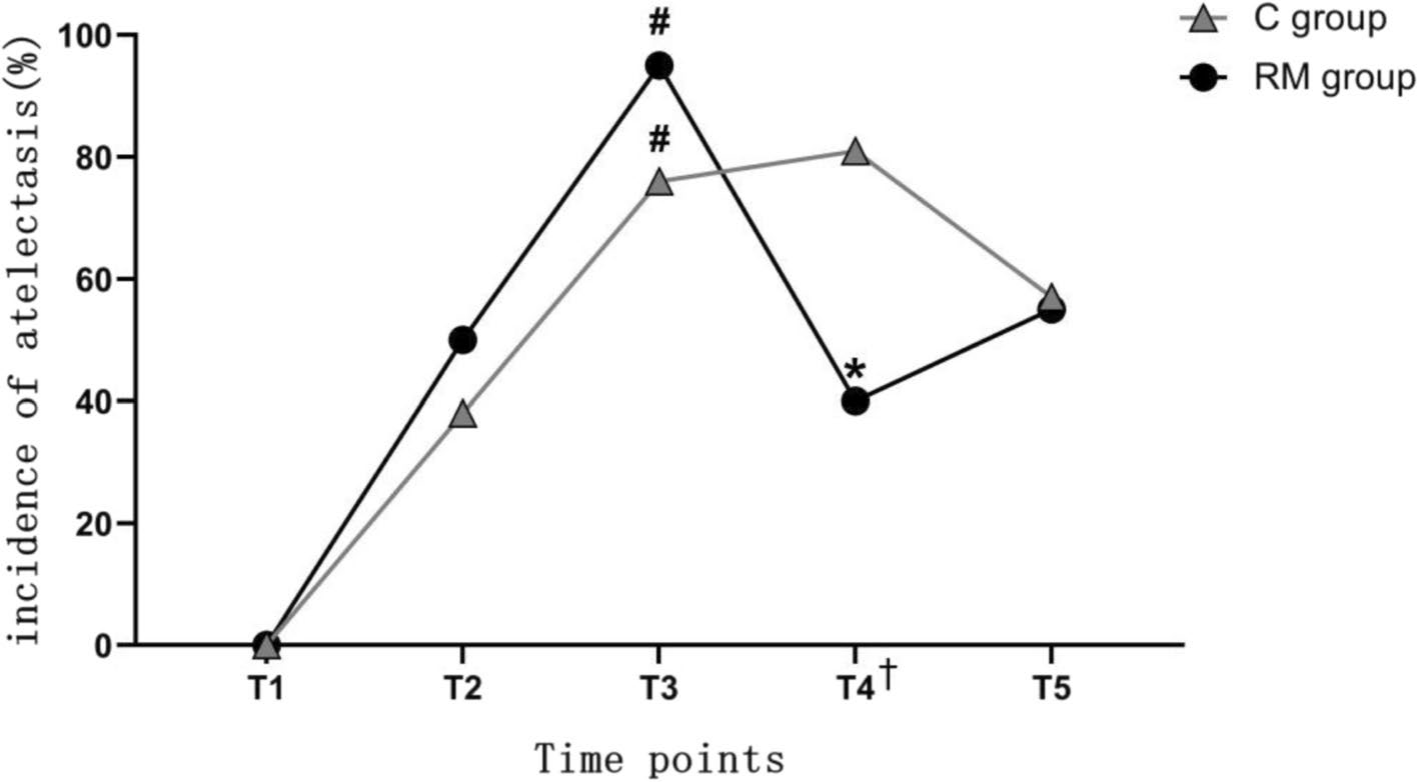
Comparison of the incidence of atelectasis at different time points in each group. T1, arrival in the operating suite; T2, 1 min after mechanical ventilation; T3, at the end of surgery; T4, 15 min after arrival in the PACU; T5, 24 h after operation. ^#^*P* < 0.05, T3 vs. T2 in the same group; *P < 0.05, T4 vs. T3 in the same group; ^†^*P* < 0.05, RM group vs. C group

The incidence of atelectasis in each group was compared at different time points, as shown in Fig. 4. Both groups experienced pulmonary atelectasis at T2, and the incidence of atelectasis was higher at T3 than at T2, with a statistically significant difference (C group, *p* = 0.008; RM group, *p* = 0.009). The incidence of atelectasis in the RM group was significantly lower in T4 compared to T3 after the last RM (*p* = 0.001), while it did not differ significantly between these two time points in the control group (*p* = 1.0). When the incidence of atelectasis at T5 was compared with that at T4, there was no significant difference in either group (C group, *p* = 0.264; RM group, *p* = 1.0). It can be seen that the incidence of atelectasis gradually increased in both groups during surgery, whereas it did not change significantly in the control group in the short term after surgery. Furthermore, it decreased in the RM group, which significantly improved pulmonary aeration (T4); however, this benefit did not last until 24 h after surgery (T5). No PPCs were observed in either group, and no side effects of recruitment maneuvers were observed in the RM group.

Supplementary Table 2 shows other characteristics of the enrolled patients that were similar between the two groups. RM did not affect oxygen saturation or pain scores at any time, nor did they significantly affect the length of stay in the PACU or hospital stay.

**Fig. 5:**
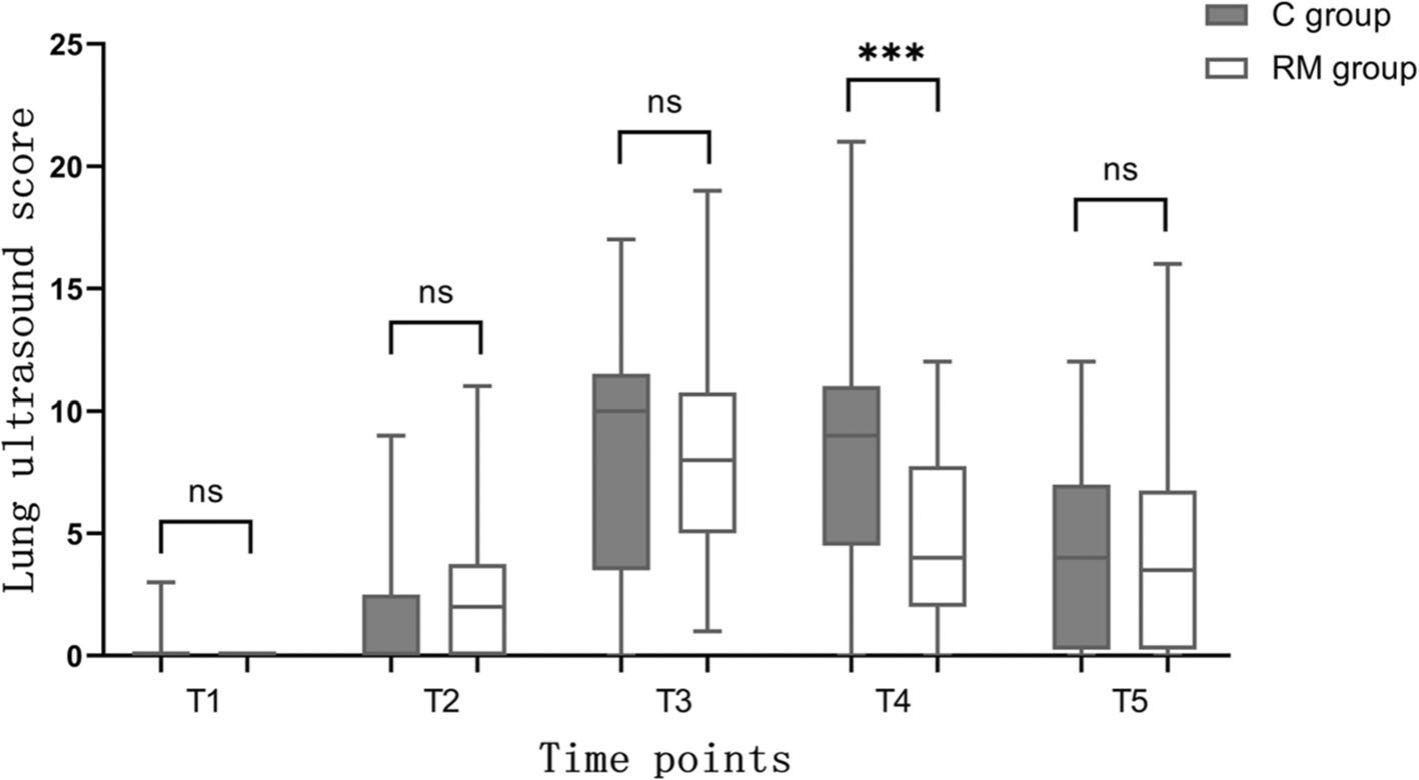
Lung ultrasound score of 12 lung regions from T1 to T5 in both groups. The box, whiskers, and bold line in the box represent the interquartile range, range, and median value, respectively. T1, arrival in the operating suite; T2, 1 min after mechanical ventilation; T3, at the end of surgery; T4, 15 min after arrival in the PACU; T5, 24 h after operation. ns, no significance; ***, *P* < 0.05

Figure 5 depicts the LUS of 12 lung regions from T1 to T5 in both groups. Only at T4, LUS were lower in the RM group compared to the C group. The difference in LUS between the two groups was similar to the difference in atelectasis incidence, which also disappeared 24 h after surgery.

**Fig. 6:**
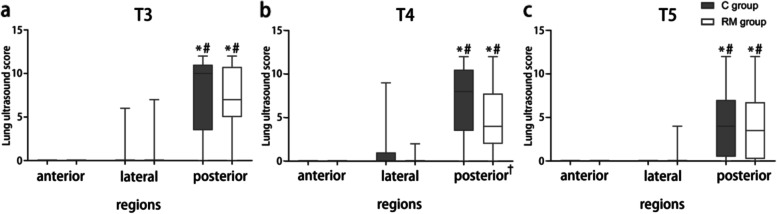
Lung ultrasound score of anterior, lateral, and posterior regions in two groups from T3 to T5. The box, whiskers, and bold line in the box represent the interquartile range, range, and median value, respectively. *P < 0.05, posterior region vs. anterior region in the same group; ^#^*P* < 0.05, posterior region vs. lateral region in the same group; ^†^*P* < 0.05, RM group vs. C group

Lung ultrasound scores of the anterior, lateral, and posterior regions in 2 groups from T3 to T5 are shown in Fig. 6 a-c. LUS of the posterior regions were higher than those of the other two regions from T3 to T5 in the same group, with no significant difference between the anterior and lateral regions. In addition, there were differences in LUS between the two groups at T4 mainly due to the difference in lung ultrasound scores in the posterior regions.

## Discussion

The combination of ultrasound-guided recruitment maneuvers and PEEP was found to reduce the incidence of atelectasis 15 min after their arrival to PACU in patients undergoing laparoscopic gynecological surgery compared to PEEP alone in this prospective randomized controlled study. However, this difference disappeared 24 h after surgery.

The incidence of atelectasis in the PACU was as high as 81.8% in the C group but was reduced to 40% in the RM group when ultrasound-guided recruitment maneuvers were used before extubation. Although the incidence of atelectasis was higher in the PACU, there was no difference in oxygen saturation or length of stay between the two groups. The following are some possible explanations. First, after surgery, patients were treated with nasal catheter oxygen inhalation, and atelectasis was effectively relieved. As a result, patients in the RM and C groups did not experience clinical symptoms such as hypoxia. Second, patients with healthy lungs have mild postoperative atelectasis, and the remaining healthy lung units can compensate for body needs; therefore, there are no clinical symptoms. Surprisingly, the difference in the incidence of atelectasis and LUS between the two groups disappeared 24 h after surgery. Furthermore, patients’ hospitalization days were not different. This could also be because these were healthy patients with no relevant comorbidities who mobilized early after surgery, which improved the control group.

Although only a few side effects of recruitment maneuvers have been reported [22, 23], which were not observed in this study, recruitment maneuvers still pose the risk of causing hemodynamic disorders and ventilator-induced lung injury [22]. As a result, ultrasound-guided recruitment maneuvers are not required for patients undergoing gynecologic laparoscopic surgery who have normal lungs.

Alveolar collapse and atelectasis recur within 5 min after general anesthesia induction and can persist postoperatively [24, 25]. In the current study, atelectasis developed after intubation and did not completely disappear 24 h later in patients undergoing laparoscopic gynecological surgery, which is consistent with the findings of previous studies [24, 25]. FIO_2_ is a critical influencing factor for atelectasis, and the incidence of atelectasis during general anesthesia for laparoscopic surgery is positively correlated with FIO_2_ levels ranging from 0.4 to 1. We used pure oxygen during induction, which may have caused an alveolar collapse in patients several minutes later [26, 27].

In particular, there was no statistically significant difference in the incidence of atelectasis during surgery between the two groups. Compared with PEEP alone, combined use of ultrasound-guided lung recruitment maneuvers and PEEP after intubation had no significant effect on intraoperative atelectasis progression. This could be because intraoperative factors are constantly causing atelectasis, and the short-term benefit of RM is not sustained or because PEEP is relatively small and does not preserve alveolar opening. In our study, atelectasis was found mainly in the posterior lung regions (the patient’s dorsum in a supine position), which corresponded to the belief that atelectasis is found mainly in gravity-dependent areas [18].

Everyone in the RM group had a second RM before extubation under ultrasound guidance until the atelectasis was completely gone. When lung ultrasound was re-examined in the PACU after extubation, atelectasis was still present in 40% of the patients, and the alveoli again collapsed rapidly. This could be due to patients lying supine in the PACU with cranial diaphragm displacement, or it could be due to other factors such as postoperative extubation, secretion obstruction, insufficient anesthetic drug metabolism, and weak respiratory motility [28].

Because ultrasound-guided RM are more effective than conventional methods in reducing atelectasis incidence in children [29], they were used to minimize its negative effects. This study also suggests that using lung ultrasound to monitor changes in lung aeration intraoperatively and postoperatively may be feasible. Furthermore, it can continuously and dynamically track changes in aeration loss, making it more widely applicable in mechanical ventilation research.

There were some limitations to this study. First, we used pure oxygen from anesthesia induction to intubation to improve anesthetic safety rather than optimal FIO_2_. The increase in FIO_2_ would contribute to the expansion of the atelectasis area [30], possibly due to accelerated absorption of alveolar gas, resulting in absorptive atelectasis. Second, because our study included patients with healthy lungs and short surgery, we expected the incidence of PPCs to be lower than after major surgery. Third, we only needed to maintain the patient at a certain depth of neuromuscular block during surgery. Although quantitative neuromuscular block monitoring was not used intraoperatively, we performed a TOF measure before extubation, ensuring reversal as previously discussed. We think that its impact on the postoperative period was limited and probably did not interfere with the trial results. However, current evidence supports that quantitative monitoring should be used whenever a muscle relaxant is administered [31]. As a result, the effect of ultrasound-guided RM on postoperative atelectasis may be negligible. Patients at high risk for PPCs should be further evaluated.

## Conclusions

In conclusion, our findings show that the combination of ultrasound-guided RM and PEEP can reduce the incidence of atelectasis in patients with healthy lungs 15 min after arrival at the PACU; however, this disappears within 24 h of surgery.

## Supplementary Information

**Additional file 1:** **Supplementary Table 1.** Incidence of atelectasis per group and per time point assessed using lung ultrasound. **Supplementary Table 2.** Other parameters of the enrolled patients.

## Data Availability

The datasets used and/or analyzed during the current study are available from the corresponding author on reasonable request.

## Abbreviations

PPCs: Postoperative pulmonary complications
PEEP: Positive end-expiratory pressure
RM: Recruitment maneuvers
PACU: Post-anesthesia care unit
BMI: Body mass index
ASA: American Society of Anesthesiologists
FIO_2_: Inspired oxygen fraction
PETCO_2_: End-tidal carbon dioxide concentration
BIS: Bispectral index
SpO2: Pulse oxygen saturation
LUS: Lung ultrasound scores
NRS: Numerical pain rating scale
