# Supplementary material for: The effect of ultrasound-guided lung recruitment maneuvers on atelectasis in lung-healthy patients undergoing laparoscopic gynecologic surgery: a randomized controlled trial
Source: BMC Anesthesiol. 2022 Jul 1;22:200. doi: 10.1186/s12871-022-01742-1 (PMC9248140; doi:10.1186/s12871-022-01742-1)
Supplement: Supplementary file 1 — Additional file 1: Supplementary Table 1. Incidence of atelectasis per group and per time point assessed using lung ultrasound. Supplementary Table 2. Other parameters of the enrolled patients. [file 12871_2022_1742_MOESM1_ESM.docx]

Supplementary Table 1. Incidence of atelectasis per group and per time point assessed using lung ultrasound.

|  | **C group, n = 21** | **RM group, n = 20** | ***P**** |
| --- | --- | --- | --- |
| T1n (%) | 0 (0) | 0 (0) |  |
| T2n (%) | 8 (38) | 10 (50) | 0.443 |
| T3n (%) | 16 (76) | 19 (95) | 0.089 |
| T4n (%) | 17 (81) | 8 (40) | 0.007 |
| T5n (%) | 12 (57) | 11 (55) | 0.890 |

We defined atelectasis to be significant if any region had a lung ultrasound score ≥2. *, comparison between the two groups in per time point, with P<0.05 considered significant. T1, arrival in the operating suite; T2, 1 min after mechanical ventilation; T3, at the end of surgery; T4, 15 min after arrival in the PACU; T5, 24 h after operation.

Supplementary Table 2. Other parameters of the enrolled patients.

|  | C group, n = 21 | RM group, n = 20 | *P** |
| --- | --- | --- | --- |
| SpO_2_(%) |  |  |  |
| T1 | 99.0(97.5–99.0) | 99.0(98.0–99.0) | 0.718 |
| T2 | 100.0(99.5–100.0) | 100.0(100.0–100.0) | 0.482 |
| T3 | 100.0(100.0–100.0) | 100.0(100.0–100.0) | 0.734 |
| T4 | 98.0(98.0–99.5) | 99.0(98.3–100.0) | 0.152 |
| T5 | 99.0(98.0–99.0) | 99.0(99.0–99.8) | 0.077 |
| PACU stay duration (min) | 44.0 ±10.7 | 41.6 ±9.2 | 0.337 |
| Hospital stay time (day) | 7.7 ±3.0 | 7.0 ±3.6 | 0.619 |

All data presented as mean ±SD or median [interquartile range] unless otherwise specified. T1, arrival in the operating suite; T2, 1 min after mechanical ventilation; T3, at the end of surgery; T4, 15 min after arrival in the PACU; T5, 24 h after operation. *, comparison between the two groups per time point, with P<0.05 considered significant.
